# Factors associated with sexual risk taking behavior by precarious urban migrants in French Guiana

**DOI:** 10.1186/s12914-018-0164-4

**Published:** 2018-06-08

**Authors:** August Eubanks, Marie Claire Parriault, Astrid Van Melle, Célia Basurko, Leila Adriouch, Claire Cropet, Mathieu Nacher

**Affiliations:** 1 Centre d’Investigation Clinique Antilles Guyane, CIC INSERM1424, Centre Hospitalier Andrée Rosemon, 97300 Cayenne, French Guiana; 2 Coordination Régionale de la lutte contre le VIH (COREVIH Guyane), Centre Hospitalier Andrée Rosemon, 97300 Cayenne, French Guiana

**Keywords:** HIV, Sexual risk, Migrants, Risk perception, French Guiana

## Abstract

**Background:**

French Guiana is highly affected by HIV. The migrant population is particularly susceptible. The objective of this study was to evaluate the level of risk of HIV transmission and its perception among migrants in French Guiana and to identify predictive factors.

**Methods:**

An HIV/AIDS Knowledge, Attitudes, Behaviors and Practices study was conducted in 2012 among migrants living in precarious neighborhoods of French Guiana.

**Results:**

Of the 1039 participants surveyed, 893 were analyzed, of which 35.6% had risky sex during the past 12 months. Sexual risk taking was higher among the migrant population than in the general population. The predictors of sexual risk taking behavior were: younger age groups, males, having a job, not living with a spouse, having first had sex before age 16, using alcohol or drugs before sex, and having engaged in commercial sex recently. The factors associated with not being aware of one’s risk were: being a woman, being from Guyana or Suriname, non-systematic use of condoms with a regular partner, and never or not recently having been tested for HIV.

**Conclusions:**

The results suggest there is still a need for information on HIV risks in a highly vulnerable population.

**Electronic supplementary material:**

The online version of this article (10.1186/s12914-018-0164-4) contains supplementary material, which is available to authorized users.

## Background

French Guiana, an overseas territory of France, is the French territory most affected by the HIV/AIDS epidemic. HIV prevalence has exceeded 1% in pregnant women for over 2 decades, and AIDS incidence is ten times higher than the national average (21.6 per 100,000 vs 2.1 per 100,000) [1, 2]. To make matters worse many persons are diagnosed late and are often lost to follow up before treatment can be properly administered. Although, the situation is described as a “generalized epidemic” there are still particularly vulnerable groups where the epidemic remains highly active. Even 30 years after the first cases appeared in French Guiana, the forces that drive this epidemic are incompletely understood [3]. Transmission is mostly reported to be heterosexual (86% compared to 39% in mainland France) but stigmatizing attitudes towards men who have sex with men may lead to underreporting [4]. The frequency of multiple heterosexual partnerships and/or concurrent relationships among men combined with a lack of consistent condom use seems to be a contributing factor [5]. Sex work, having tried crack cocaine and being a migrant are additional risk factors in French Guiana [3]. Over 75% of persons living with HIV in French Guiana are of foreign nationality. The exact reason for this is not clear. A first hypothesis is that migrants come from areas of high prevalence and concentrate high risk groups that thus acquire HIV outside of French Guiana; another complementary hypothesis is that migration and poverty constitute a context where sexual risks increase. In this context the objective of the present study was to evaluate the level of risk of HIV transmission among poor urban migrants in French Guiana and to identify predictive factors. A secondary objective was to evaluate risk perception and its predictors among migrants taking sexual risks in French Guiana.

## Methods

### Study design

#### Setting and participants

Data collected by the Clinical Investigation Center Antilles-Guyane during a KABP study of HIV and migrants in 2012 was used. It consisted of a multicentric observational cross-sectional study carried out French Guiana. The target population was sexually active migrants in French Guiana in underprivileged neighborhoods in one of the 3 main cities -Cayenne, Kourou, and Saint Laurent du Maroni.

#### Inclusion criteria

Only migrants were included in this study. A migrant is considered to be a foreign person, born abroad and residing in France, including French overseas territories. This definition of immigrants is used by the High Council of Integration and INSEE for their surveys. Only migrants 18 years or older and accepting to participate in the study were interrogated. Only participants engaging in sexual intercourse within the last 12 months of the study were included in the statistical analysis.

#### Sampling

For this study, non-probabilistic sampling was used. Participants were chosen from neighborhoods defined as precarious, presenting at least one of the following three characteristics: no access to running water, no access to electricity, or not benefiting from municipal garbage collection. This information was obtained from the prefectoral authorities who also provided population size estimates and maps. In practice, the population in these neighborhoods mostly consists of immigrants, who usually spontaneously aggregate by country of origin. This detailed information on precarious urban areas is compiled by the municipalities and the prefectoral authorities for the purposes of urban development planning and social services development. Once these areas were identified the surveyors were sent to the neighborhoods on different days of the week and different hours of the day in order to ensure greater diversity. They were assisted by local mediators who know the population in order to facilitate the survey. The surveyors started at the center of the neighborhood anda random direction was determined with the aid of a spinner. They then walked in the randomly selected direction and knocked at one of every two housesand then, in each selected house, verified that the person was a migrant, and used the shortest straw method to select the surveyed individual. If the house was empty they came back twice. They did so until the end of the street and then came back to the center of the neighborhood where they selected another random direction and reiterated the above. The number of individuals surveyed was proportional to the neighborhood population size given by the prefectoral authorities. For selected participants, the study was a knowledge, attitudes, behavior and practices study (based on questionnaires by Family health international’s behavioral surveillance surveys). Among the questions, one was about sexual activity in the past 12 months, a question which allowed us to select the subgroup of persons having had sex within the past year.

#### Ethics and funding

The study was approved by the Comité d’Evaluation Ethique de l’Inserm (CEEI) (IRB N°12–057), the Comité Consultatif pour le Traitement de l’Information en matière de Recherche sur la Santé (CCTIRS) and the Commission Nationale Informatique et libertés (CNIL) (DR-2012-261). The study was funded by the Program INTERREG Caraibes IV, Fond Européen de Développement Régional (FEDER), the University Hospital of Pointe-à-Pitre, Cayenne Hospital, and AIDES.

### Statistical methods

#### Data collection

The data were collected from an anonymous structured questionnaire of 108 questions, translated into five languages (French, English, Portuguese, Spanish and Haitian Creole) and administered in an individual interview setting. After the interview, a prevention kit was provided that included male and female condoms, lubricant gel, an information brochure on HIV and STIs in the context of French Guiana, and a gift coupon to compensate for the time spent on answering questions. The study was based on BSS questionnaires produced by Family Health International and followed their criteria. This KABP study was delivered by trained local interviewers specialized in administering surveys to vulnerable populations.

#### Conceptual framework

This study aimed to investigate two outcome variables: risky sexual behavior and perception of risk. Our analysis concerned migrants who were recently sexually active, meaning they were sexually active within the last twelve months. Using the 12-month reference period and the last sexual encounter were considered useful for capturing the most recent behavior and minimized recall errors [6]. After reviewing the literature, our hypothesis was that the following risky sexual behaviors were associated with increased susceptibility to HIV infection: non-systematic use of condoms with casual or commercial sex partners, having had multiple sexual partners and having engaged in concurrent partnerships. Multiple and concurrent partnerships are common in French Guiana and are a known driver of the HIV/AIDS epidemic [5]. In countries with little condom use, and high levels of multiple and concurrent sexual partners, individual sexual history is largely used to define HIV risk [7].

Due to the context in French Guiana, the high prevalence of HIV, and the associated risk, condom use was interpreted strictly as using condoms systematically (every time) or non-systematically (almost every time, sometimes, or never). This definition took into account the usage of condoms during the last sexual encounter with a casual or commercial partner as well as the frequency of condom use with these partners. Regular partners were defined as partners with whom participants had sex for at least 6 months. Commercial partners were defined as partners with whom participants bought or sold sex in exchange for money, services, goods or drugs. Casual sex partners were defined as partners who were not “regular” or “commercial.” Multiple partners meant the individual had more than one sexual partner during the last year. A concurrent relationship was defined as having had more than one partner simultaneously.

The dependent binary variable, “risk”, was created, coding 1 if the individual had engaged in risky sex defined as non systematic condom use with casual or commercial partners, or had multiple sexual partners or engaged in concurrent partnerships Otherwise they were coded as 0 and placed in the “no risk” group. The independent variables were chosen as potential HIV risk factors after reviewing the available literature.

A second outcome variable was defined to further explore individual’s HIV risk perception in those who were considered to have sexual risk. This was evaluated using a question asking “How would you rate your own risk of getting HIV?” The answers could be high risk, low risk, does not know or no response.

#### Statistical analysis

The statistical analysis was performed using Stata© 13.0 (Stata Corp LP, College Station, TX, USA).

##### Descriptive and comparative

A descriptive analysis was first performed. Then a bivariate analysis was performed to compare potential predictive factors between groups; Qualitative variables were compared using a χ^2^ test or a χ^2^ test for linear trend. Variables were considered as significantly associated with risk if their *p* value was < 0.05.

##### Multivariate analysis

Given that the event “risk” was frequent (> 10%), the common approximation of risk ratios by odds ratios would have led to distorted estimates. We used a modified Poisson regression instead to avoid this problem [8] and obtain prevalence ratios (PR) instead. The variables chosen for the multivariate model were those with a *p* value< 0.10 in the comparative analysis. A backwards stepwise model was used, simultaneously inserting all variables with a *p-*value < 0.10 and removing the variable with the highest p-value each time. Only variables significant at the 5% threshold were retained. Pearson’s Goodness-of-fit test was applied. Variance inflation factors were used to search for colinearity between covariates (value < 4). A similar procedure was used for risk perception.

## Results

There were 1039 participants of which 893 reported having had sex in the past 12 months and were retained for analysis in this study. Overall, using our composite risk definition, 35.6% of the study population had risky sex during the past 12 months (Table 1) Additional file 1.

**Table 1:** Description of the sample of migrants studied

Variable	French Guiana n(%)
Engages in risky sexual behavior	893
Takes no risks	575 (64.4)
Takes risks	318 (35.6)
Age	
[mean +/−sd]	[35.4 +/− 10.8]
[95% Conf. Interval]	[34.7–36.1]
Sex	893
Female	536 (60.0)
Male	357 (40.0)
Type of employment	892
Has no job	432 (48.4)
Has a work contract (temporary or long-term)	136 (15.3)
Works without a contract	304 (34.1)
Is a business owner	20 (2.2)
Nationality	893
French	14 (1.6)
Guyanese	119 (13.3)
Surinamese	196 (22.0)
Dominican (Dominican Republic)	46 (5.2)
Brazilian	231 (25.9)
Haitian	262 (29.3)
Dominican (Dominica)	3 (0.3)
Saint Lucien	1 (0.1)
Other	21 (2.4)
Marital status	893
Married, living with spouse	136 (15.2)
Married, living with another sex partner	11 (1.2)
Married, living alone	15 (1.7)
Not married, living with regular sex partner	340 (38.1)
Not married, not living with sex partner	388 (43.5)
Other	3 (0.3)
Immigration Status	870
Undocumented (no residency permit)	314 (36.1)
Temporary (3 month/1 year permit, refugee)	318 (36.6)
Longterm (10 year residency card)	238 (27.4)
Age at first intercourse *	862
[mean +/−sd]	[15.9 +/− 3.1]
[95% Conf. Interval]	[15.7–16.1]
HIV test	892
No, never had an HIV test	113 (12.7)
Yes, has had an HIV test	779 (87.3)
History of drug use	893
Never tried any drugs	646 (72.3)
Yes, tried at least once	247 (27.7)
Recent alcohol abuse	890
No, not in the past month	551 (61.9)
Yes, > 5 drinks in one day in the past month	339 (38.1)

Overall, 34.7% of men (124/357) and 12.7% of women (68/535) declared multiple concomitant sex partners. Regarding transactional sex, 15% of men (54/355) declared having solicited transactional sex in the past 12 months, and 8.2% of women (44/536) and 2% (8/395) of men declared having received money or drugs in exchange for sexual relations. Regarding the last sexual relation with a commercial partner, 7.1% of men (4/56) and 4.6% of women (2/43) declared they had not used a condom. Regarding the last sexual relation with a casual partner 17% of men (21/123) and 28% of women (26/96) declared they had not used a condom. Regarding the last relation with a regular partner, 31.9% of men and 23.3% of women used a condom.

In comparison, a 2011 study in the general population of French Guiana showed that 14% of men and 1.1% of women had concurrent multiple sex partnerships [9]. In the same study, 4.6% of men declared having transactional sex during the past 12 months in the general population of French Guiana. For women, the exact value was only available for Martinique, Guadeloupe and French Guiana and was 0.3% with no significant difference between territories. Regarding condom use at the last intercourse with a commercial sex partner in French Guiana, 4.5% of men in the general population did not use a condom. The aggregated data in the KABP study report [9] made further comparisons impossible.

**Fig. 1:**
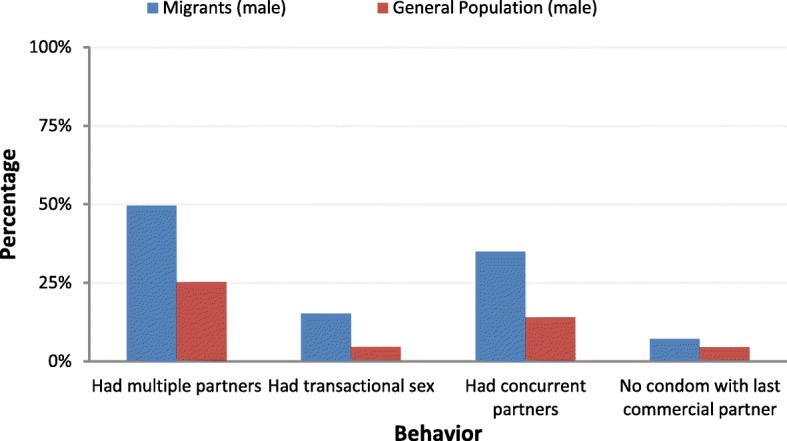
Comparison of sexual risks between males surveyed in the general population and in migrants

Figure 1 compares these risks between males surveyed in the KABP study in the general population [9] and our study.

Table 1 also shows the details of the study population in French Guiana. Overall 65% of men (258/397) had partners of a different nationality before (126/397 (32%) regularly and 132/397 (33%) occasionally). For women, 58% (371/642) had sex partners of a different nationality before (200/642 (31%) regularly, and 171/642 (26.6%) occasionally).

Table 2 shows that younger age groups, males, persons working with or without contract, persons who were not married, or were married but living with another sex partner, persons having first had sex before age 16, persons using alcohol or drugs before sex, and persons having engaged in commercial sex recently were more likely to have had risky sex. Those engaging in high risk were also more likely to be aware of being at risk.

**Table 2:** Univariate and Multivariate Analysis, the association between different variables and the two groups: risk and no risk, in French Guiana with the aid of a modified Poisson regression, *n* = 815

Variable	Univariate analysis	Multivariate analysis	
	Unadjusted PR [95% CI]	Adjusted prevalence ratio [95% CI]**	*p* value
Age Group			
18–29	1	1	–
30–39	0.77 [0.63–0.93]	0.88 [0.73–1.05]	0.161*
40–50	0.51 [0.38–0.68]	0.65 [0.51–0.82]	< 0.001
> 50	0.61 [0.42–0.87]	0.66 [0.47–0.91]	0.013
Sex			
Female	1	1	–
Male	2.04 [1.70–2.43]	1.60 [1.34–1.91]	
Type of employment			
Has no job	1	1	–
Has a work contract	1.72 [1.30–2.27]	1.46 [1.12–1.91]	0.006
Works without a contract	2.41 [1.97–2.96]	1.58 [1.29–1.94]	< 0.001
Is a business owner	1.35 [0.68–2.70]*	1.15 [0.59–2.24]	0.692*
Marital status			
Married, living with spouse	1	1	–
Married, living with another sex partner	4.50 [1.71–11.81]	3.68 [1.84–7.35]	< 0.001
Married, living alone	3.30 [1.20–9.08]	1.92 [0.75–4.89]	0.171
Not married, living with regular sex partner	3.20 [1.77–5.80]	2.10 [1.21–3.65]	0.008
Not married, not living with sex partner	6.66 [3.75–11.83]	3.65 [2.12–6.27]	< 0.001
Other	8.24 [3.09–21.98]	6.04 [2.76–13.22]	< 0.001
Pendular migration			
No risk	1	1	–
Low risk	2.84 [2.44–3.31]	1.46 [1.19–1.78]	0.001
High risk	1.23 [0.85–1.80]*	1.16 [0.80–1.68]	0.424*
Engaged in commercial sex recently			
No	1	1	–
Yes	3.57 [3.18–4.0]	2.08 [1.77–2.46]	< 0.001
Age at first intercourse			
< 16	2.40 [1.96–2.93]	1.53 [1.27–1.86]	< 0.001
≥ 16	1	1	–
Don’t know, no response	1.51 [0.88–2.59]*	1.57 [0.99–2.49]	0.055*
Use of drugs or alcohol before/during sex			
No	1	1	–
Yes	2.96 [2.55–3.45]	1.30 [1.08–1.56]	0.006
Perception of risk to contract HIV			
Low Risk	1	1	–
High Risk	1.89 [1.49–2.39]	1.41 [1.11–1.80]	0.005
Don’t Know	1.62 [1.34–1.97]	1.18 [0.97–1.44]	0.102*

*non-significant, *p* value > 0.05

**CI Confidence Interval

Table 3 shows that in terms of risk perception among those taking risks, the factors independently associated with not being aware of one’s risk were: being a woman, being from Guyana or Suriname, non-systematic use of condoms with a regular partner, and never having been tested for HIV.

**Table 3:** Factors associated with not perceiving one’s risk in those actually having sexual risks

Variable *n* = 261	Risk with perception *n* = 232 n(%)	Risk with no perception *n* = 86 n(%)	Adjusted prevalence ratio [CI 95%], P	
Sex				
Female	90 (38.8)	45 (52.3)	1	–
Male	142 (61.2)	41 (47.7)	0.66 [0.44–0.98]	0.040
Nationality				
Guyanese	23 (9.9)	14 (16.3)	2.39 [1.31–4.35]	0.004
Surinamese	48 (20.7)	36 (41.9)	1.92 [1.09–3.38]	0.025
Brazilian	81 (34.9)	24 (27.9)	1	–
Use of condoms with regular or trusted partner				
Non-systematic	118 (61.1)	58 (84.1)	1	–
Systematic	75 (38.9)	11 (15.9)	0.43 [0.24–0.78]	0.006
Last HIV test				
Never	35 (15.2)	17 (19.8)	1.93 [1.18–3.15]	0.009
Not recently	51 (22.1)	34 (39.5)	1.62 [1.02–2.60]	0.043
Recent	145 (62.8)	35 (40.7)	1	–

## Discussion

The present study showed that over a third of migrants had been involved in risky sex (non-systematic use of condoms with casual or commercial sex partners, and/or had multiple sexual partners and/or engaged in concurrent partnerships) in the past 12 months. There was a greater proportion of persons involved in concurrent sexual partnerships among migrants than in the general population of French Guiana. Paying or receiving money for sex was more frequent for both males and females in the migrant population than in the general population of French Guiana. Over half of the migrant population declared having sex partners of another nationality, suggesting that the epidemic may progress between different communities. Increased sexual risks in migrants have been described in several studies worldwide and French Guiana is no exception [10–12]. Isolation, the ensuing decrease of community regulation of sexuality, vulnerability and poverty converge to increase the migrants’ likelihood of engaging in sexual risks.

The study of the predictive factors associated with risky sex showed that younger age groups, males, having a job (but not being a business owner), not living with a spouse, having first had sex before age 16, those using alcohol or drugs before sex, and those having engaged in commercial sex recently were more likely to have had risky sex. Among those who were at risk, women, persons from Guyana or Suriname, those who did not systematically use condoms with their regular partner, and who had never, or not recently, been tested for HIV were less likely to be aware of being at risk. According to the health belief model, changing behavior requires feeling potentially at risk. Thus the persons who do not use condoms or had not done any HIV test presumably because they did not feel at risk. This suggests there is still a need for information on HIV risks.

This study has several limitations. The cross-sectional design was a potential source of confounding and biases. Sampling was non-probabilistic; therefore results cannot be generalized for the entire migrant population in French Guiana. In addition, migrants were chosen from precarious neighborhoods, which could have over-represented socially vulnerable groups. Declarative data can be problematic as well because participants may not always remember or be inclined to reveal intimate behavior. In addition, there may have been cultural or gender differences in the propensity to reveal certain behaviors [5–7]. Transmission may occur with a regular partner, and our definition of risk was focused on casual and commercial partners, and may have underestimated the risk of getting infected with HIV with the regular partner.

Despite these limitations, this study is the first to describe and analyze predictors of sexual risk in the precarious migrant population of French Guiana.

The reason why such a large proportion of persons living with HIV in French Guiana are migrants is not clear and may result from the high prevalence in the persons’ countries of origin and the arrival of already infected persons in French Guiana. Recent UNAIDS data suggests the adult prevalence between 15 and 49 years in the main immigrant groups (Haiti, Suriname, Guyana) was respectively 1.7, 1.1, and 1.5% versus 1.6% in French Guiana using Spectrum and AIM, which gives some plausibility but no definitive proof to the first hypothesis [10, 13]. In Europe, between 2002 and 2014, the proportion of infections acquired in European countries in non-MSM Africans with HIV was variable and ranged between 2 and 37% [14]. Although methodological differences complicated the comparisons, the studies converged in the conclusion that there is ongoing sexual transmission after the arrival in the host country. However, although French Guiana is technically an overseas territory of Europe, the epidemic and the migration patterns are probably different from the patterns observed in Europe. Following the seroprevalence of migrant cohorts over time, which is challenging given their mobility, would yield more information on the respective importance of “imported” and locally acquired HIV infections among migrants. Methods based on CD4 count decline and date of arrival were used in French Guiana and suggested that over half of HIV-infections among migrants were acquired after arriving in French Guiana [15, 16]. These methods tend to yield a larger estimated proportion of post-migration HIV acquisition than studies based on clinic reports. These recent estimates [16] are coherent with the present observation of frequent risky sexual behavior among migrants in French Guiana.

## Conclusion

The present study showed a third of the surveyed migrants living in the most precarious areas had risky sexual behavior which thus presumably contributes to driving the epidemic among migrants in French Guiana. Younger age groups, males, persons having a job, not living with a spouse, having first had sex before age 16, using alcohol or drugs before sex, and those having engaged in commercial sex recently were more likely to have had risky sex. In the French Guianese context of an epidemic that mostly involves migrants, where access to care is often problematic for the most vulnerable populations, this is information should guide prevention and testing efforts.

## Additional file

Additional file 1: **Table S1.** Breakdown of risky behavior, comparison between the general population in French Guiana and the migrant population in French Guiana (DOCX 15 kb)

## Abbreviations

AIDS: Acquired Immune Deficiency Syndrome
CCTIRS: Comité Consultatif du Traitement de l’Information en matière de Recherches en Santé
CEEI: Comité d’évaluation Ethique de l’INSERM
CNIL: Commission Nationale Informatique et Libertés
FEDER: Fonds Européen de Développement
HIV: HumanImmunodeficiency Virus
INSEE: Institut National de la Statistique des Etudes Economiques
INSERM: Institut National de la Santé et de la Recherche Médicale
KABP: Knowledge, Attitudes, Behaviors, and Practices
MSM: Men who have Sex with Men
