# Supplementary material for: Factors associated with sexual risk taking behavior by precarious urban migrants in French Guiana
Source: BMC Int Health Hum Rights. 2018 Jun 8;18:24. doi: 10.1186/s12914-018-0164-4 (PMC5994113; doi:10.1186/s12914-018-0164-4)
Supplement: Supplementary file 1 — Table S1. Breakdown of risky behavior, comparison between the general population in French Guiana and the migrant population in French Guiana (DOCX 15 kb) [file 12914_2018_164_MOESM1_ESM.docx]

**Additional file 1 Table S1 Breakdown of risky behavior, comparison between the general population in French Guiana and the migrant population in French Guiana.**

| **Variable (%)** | **General population***  n = 1558 | | **Migrant population**  n = 893 (357M, 535 F) | |
| --- | --- | --- | --- | --- |
| Sex(M=male, F=female) | **M** | **F** | **M** | **F** |
| Had multiple partners | 25.3 | 7.0 | 49.6 | 23.4 |
| Engaged in commercial sex | 4.6 | 0.3** | 15.2 | 8.2 |
| Had a concurrent relationship | 14 | 1.1 | 34.9 | 12.7 |
| No condom with last commercial partner | 4.5 | - | 7.1 | 4.6 |
| No condom with last occasional partner** | - | | 17.2 | 28.9 |
| Non-systematic use of condoms with commercial partners** | - | | 8.93 | 13.95 |
| Non-systematic use of condoms with occasional partners** | - | | 22.8 | 41.8 |

- *data from KABP 2011 Antilles Guyane, [37]
- ** exact value for French Guiana not available, value for women in all FAD
